# Supplementary figures and images for: Determination of the reference genes for qRT-PCR normalization and expression levels of MAT genes under various conditions in Ulocladium
Source: PeerJ. 2020 Nov 23;8:e10379. doi: 10.7717/peerj.10379 (PMC7690293; doi:10.7717/peerj.10379)

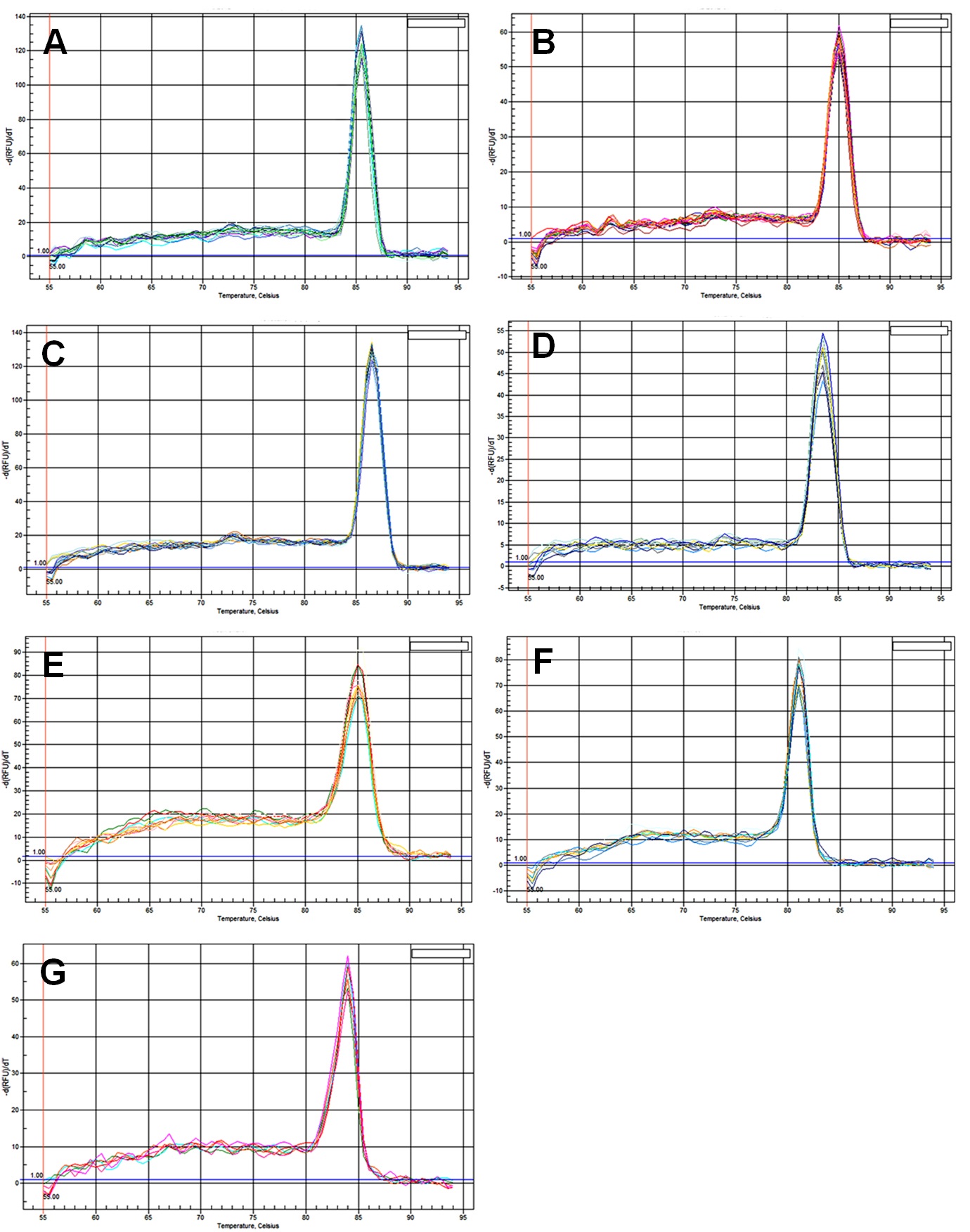

Supplement: Supplemental Information 2 — A:Actin; B:β-tubulin; C:EF-1α; D:GAPDH; E:RL13; F:TBP; G:UBC. [file peerj-08-10379-s002.jpg]
